# Hypertension in the very old; prevalence, awareness, treatment and control: a cross-sectional population-based study in a Spanish municipality

**DOI:** 10.1186/1471-2318-9-16

**Published:** 2009-05-08

**Authors:** Alba Aguado, Flora López, Sonia Miravet, Pilar Oriol, M Isabel Fuentes, Belén Henares, Teresa Badia, Lluis Esteve, Javier Peligro

**Affiliations:** 1 CAP Sagrada Familia, Consorci Sanitari Integral, Barcelona, Spain; 2 Catalan Institute of Health, ABS Martorell, Martorell, Spain

## Abstract

**Background:**

Information on hypertension in the very elderly is sparse. Until recently evidence of benefits from pharmacological treatment was inconclusive. We estimated the prevalence of hypertension in subjects aged 80 or more, the proportion of awareness, treatment and control. Explanatory variables associated with good control were also studied.

**Methods:**

Cross sectional, population-based study, conducted in Martorell, an urban Spanish municipality, in 2005. By simple random sampling from the census, 323 subjects aged 80 or more were included. Patients were visited at home or in the geriatric institution and after giving informed consent, the study variables were collected. These included: supine and standing blood pressure and information about diagnosis and treatment of hypertension. The estimation and 95% confidence interval were obtained and a logistic regression model was used to study explanatory variables associated with blood pressure below 140/90 mm Hg.

**Results:**

The prevalence of hypertension was 72.8% (95%CI: 69.5 – 76.6%) and 93% of the patients were aware of this condition, of whom 96.3% (95%CI: 93.65 – 97.9%) had been prescribed pharmacological treatment and 30.7% (95%CI: 25.8 – 36.1%) had blood pressure below 140/90 mm Hg. Some of the patients (43%) had one antihypertensive drug and 39.5% had two in combination. Explanatory variables associated with blood pressure below 140/90 mm Hg included prescription of a diuretic, OR: 0.31 (95%CI: 0.14 – 0.66), and history of ischemic heart disease, OR: 0.21 (95%CI: 0.1 – 0.47).

**Conclusion:**

The prevalence of hypertension in population aged 80 or more was over 70%. Most patients were aware of this condition and they had antihypertensive medication prescribed. Approximately one third of treated patients had blood pressure below 140/90 mm Hg. Patients with heart disease and with diuretics had more frequently blood pressure below this value.

## Background

In Spain 655,924 men and 1,247,295 women were 80 years of age or more in 2005, which represents 3.8% and 5.5% of total male and female Spanish population [1]. Life expectancy at age 80 was 9.2 years for women and 7.6 for men [2]. An increase of this age group is expected because of the progressive aging of the population and the increase in life expectancy. Cerebrovascular diseases are the main cause of death for this age group and arterial hypertension is the principal risk factor for this condition. In several studies the prevalence of hypertension in Spanish geriatric population ranged from 65% to 76%, and they include people aged over 60 years [3,4], 65 [5] or 75 [6].

Until very recently evidence that treating patients 80 years of age or older is beneficial was inconclusive [7-9]. Most clinical trials on hypertension conducted in geriatric population included a low proportion of people aged 80 or more [10-14]. In 2008 the results of HYVET clinical trial were published [15]. For the first time benefits of active treatment (indipamide with or without perindopril) were proved for the very elderly.

In our country there is little information available about hypertension in the very elderly. The objectives of the study were to estimate the prevalence of hypertension in subjects aged 80 years or more, awareness, treatment and control. Explanatory variables associated with good control were also studied.

## Methods

### Setting and subjects

The study is cross sectional, population-based and it was conducted in 2005 in Martorell. It is an urban municipality close to Barcelona, Spain, with 25,718 inhabitants (879 aged 80 or more). Inclusion criteria were people aged 80 years or more, living at home or in geriatric institutions, included in the census of the municipality who gave informed consent to participate in the study. Exclusion criteria included disorders that made impossible to measure blood pressure, extreme mobility alteration and terminal patients.

By simple random sampling 323 subjects were selected. Sample size was calculated assuming a proportion of 0.7, with 0.05 precision and a confidence level of 95%. Lost subjects were replaced.

### Data retrieval and statistical analysis

Randomized patients were contacted and visited at home or in their geriatric institution by a nurse or physician, where data were collected. Except for the first 10 patients visited by a nurse, the subjects were visited by physicians, all of them members of the research team. Measurements were standardized. Patients who gave informed consent were included in the study. Variables studied were: Demographic (age, gender, residence), Diagnostic blood pressure (DBP) for those who were not previously diagnosed hypertension, control blood pressure (CBP) for previously known hypertensive patients, standing blood pressure (to detect orthostatic hypotension), prescribed antihypertensive medication, cardiovascular risk factors (tobacco, hypercholesterolemia, type 2 diabetes mellitus) and cardiovascular disease (cerebral vascular disease, coronary disease, peripheral vascular disease). The Barthel index was used to rate ability to perform activities of daily living. It is a 10 item scale with a highest possible score of 5 to 15 points on each item [16]. It gives a total score from 0 (full dependence) to 100 (independence).

Blood pressure was ascertained under standardized conditions. After a 5 minute-rest blood pressure was measured in the seated position, using appropriately sized cuffs and calibrated and validated automatic devices (HEM-711, OMRON). Feet were flat on the floor and arm supported at heart level. Contralateral arm was verified.

Measurement of Diagnostic blood pressure: patients who were not previously diagnosed hypertension had two measurements separated one another more than 2 minutes, in three different days at least 7 days apart. Diagnostic blood pressure was the mean value. For blood pressure measurements and criteria for definition of hypertension we followed the hypertension guidelines of the Catalan Society of Family and Community Medicine [17]. These criteria are similar in most international guidelines. The patient's physician was informed about new diagnosis of hypertension. Research carried out was in compliance with the Helsinki Declaration. The project was aproved and granted by the research board of the Catalan Society of Family and Community Medicine.

Measurements of Control blood pressure: patients who were aware of this condition had two blood measurements separated one another more than 2 minutes. Mean value was considered.

Hypertension was defined as systolic blood pressure greater than or equal to 140 mm Hg or diastolic blood pressure greater than or equal to 90 mm Hg, or receiving medication specifically for the indication of hypertension. Control was considered acceptable when systolic blood pressure was less than 140 mm Hg and diastolic blood pressure less than 90 mmHg among treated hypertensive patients.

Blood pressure was also measured in the standing position. Orthostatic hypotension was considered when a reduction of at least 20 mm Hg in systolic blood pressure or at least 10 mm Hg in diastolic blood pressure was observed within 3 minutes of standing up [18]. Pulse pressure was considered as the difference between systolic and diastolic blood pressure.

The estimation and 95% confidence interval were obtained for the prevalence of hypertension, awareness and control. Logistic regression models with forward stepwise selection of variables were used to study explanatory variables associated with blood pressure below 140/90 mm Hg. Independent variables studied were age, gender, functional status, residence at home or in geriatric institutions and cardiovascular disease.

## Results

### Description of studied population

A total of 447 individuals aged 80 and over were randomly selected from the municipal census. The following did not participate in the study for the following reasons: 48 had already died, 35 refused, 30 could not be contacted, 9 did not live in the municipality and 2 because of severe dementia. Response rate was 81% (399 people were contacted and 323 were included).

The total number of subjects included was 323. The percentage of women was 62.5%, mean age 84.9 (S.D: 3.6). Most of them lived at home (91.6%) and 8.4% lived in geriatric institutions. According to their functional status 44.3% were independent (scored 100 in Barthel index) and 41.2% had mild dependence (scored 60 to 99).

The prevalence of other cardiovascular risk factors was: diabetes mellitus type 2 (20.7% of the subjects), hyperlipidemia (35%) and 3.4% were current smokers. The prevalence of ischemic heart disease was 13%, stroke 17.3% and peripheral vascular disease 11.1%.

### Hypertension: prevalence and management

In table 1, total prevalence of hypertension is presented as well as the percentage of patients who were aware of it, had pharmacological treatment and had blood pressure below 140/90 mm Hg. The number of active ingredients prescribed to patients who were diagnosed hypertension was: 0 (3.7%), 1 (43.1%), 2 (39.5%), 3 (13.5%) and 4 (0.3%). In tables 2 and 3 the prescription of main therapeutic groups and combinations is presented.

**Table 1:** Prevalence of hypertension, awareness, treatment and control in a population-based study conducted in a Spanish municipality with patients aged 80 and over.

	**Percentage**	**95% CI-lower**	**95% CI-upper**
Total prevalence of hypertension	72.8	69.5	76.6

Awareness	92.7	89.5	95.1

Pharmacological treatment	96.3	93.6	97.9

Blood pressure below 140/90 mmHg	30.7	25.8	36.1

Total prevalence of hypertension, awareness (out of total hypertension), treatment (out of diagnosed patients) and blood pressure below 140/90 (out of diagnosed patients).

**Table 2:** Prescription of therapeutic groups in patients aged 80 and over in a Spanish municipality.

**Treatment**	**Percentage**	**95% CI-lower**	**95% CI-upper**
Diuretic	68.1	60.6	71.1

ACE inhibitor	41.3	35.9	46.9

Calcium antagonist	23.4	19	28.4

Angiotensin II antagonist	23.4	19	28.4

Beta blocker	7.3	4.9	10.8

Alpha1 blocker	4.6	2.8	7.5

Others	0.9	0.3	2.7

Percentage of patients with prescription of the main pharmacological groups.

ACE-Inhibitor: angiotensin-converting enzyme inhibitor.

**Table 3:** Prescription of different combinations and monotherapies in patients aged 80 and over in a Spanish municipality.

**Treatment**	**Percentage**	**95% CI-lower**	**95% CI-upper**
Diuretic + ACE inhibitor	18.4	14.4	23.1

Diuretic	13.8	10.4	18.1

ACE inhibitor	12.4	9.2	16.5

Diuretic + AA	10.6	7.6	14.5

Calcium antagonist	7.4	14.9	10.8

AA	6.9	4.6	10.3

Diuretic +ACE-I +calcium antagonist	6.5	4.2	9.7

Diuretic + calcium antagonist	5.1	3.1	8.1

Diuretic + beta blocker	2.8	1.4	5.2

Diuretic + AA + calcium antagonist	2.3	1.1	4.6

Others	13.8	9.9	19.0

Most common pharmacological combinations prescribed.

ACE-I: angiotensin-converting enzyme inhibitors. AA: angiotensin II antagonists.

The percentage of patients with hypertension diagnosis with systolic blood pressure below 140 mm Hg and diastolic pressure below 90 mmHg was 30.7% (95% CI: 25.8 – 36.1%). Explanatory variables associated with blood pressure below 140/90 mm Hg: there was good control in 35.4% of patients who were prescribed a diuretic and in 21.6% of patients without this medication (OR: 0.31; 95%CI: 0.14 – 0.66). The percentage of patients with ischemic heart disease with good control was higher (57.6%) than that of patients without this condition (26.6%), odds ratio: 0.21 (95%CI: 0.1 – 0.47). Age and sex were not statistically significant.

Pulse pressure was 65 mm Hg or higher in 65% of subjects (95% CI: 60.5 – 69.3%); in 72.3% (95% CI: 67.3 – 76.9%) of patients with hypertension and in 45.4% of people without this condition (95% CI: 60.5 – 69.3%). Orthostatic hypotension was present in 9.6% of patients (95% CI: 7.2 – 12.6%); in 8.3% (5.7 – 11.9%) of patients with hypertension and 12.4% (8–18.7%) of patients without it.

## Discussion

The prevalence of hypertension in this population aged 80 or more is very high, it exceeds 70% and most of the patients (93%) are aware of this condition. Most subjects known to be hypertensive have pharmacological treatment (96%), even though at the time data were collected there was no clear evidence of the benefits in this age group. However, only 31% of the patients aware of this condition had blood pressure below 140/90 mmHg.

Another study in elderly population conducted in Spain has a similar prevalence of hypertension [3]. However, awareness and treatment is higher in our study. Banegas who included patients over 60 found a percentage of awareness of 65% [3], (while ours was 92%) and 55.3% of treatment (96% in our study). The reason might be because in our study the subjects are older, and the very elderly might have more contact with the health system and so hypertension could be more easily detected and treated. Martinez Pastor studied patients aged over 75 controlled in primary care in Spain, and 92% were prescribed antihypertensive treatment [6].

The very old have been under-represented in many past randomized clinical trials [8]. As a result there is a lack of evidence of the benefit of many pharmacological treatments addressed to this age group. The results of HYVET, a clinical trial enrolling hypertensive patients aged 80 or more, have been published recently [15]. The benefit of active treatment (indipamide with or without perindopril) for the very elderly has been proved for the first time. However, when we obtained the data for the study this information was not available. Considering the lack of evidence clinicians had about the benefits of treatment, it is interesting to point out that most of the patients had antihypertensive medication prescribed and more than half of them had a combination of at least two drugs. Probably the patients started medication when they were younger and they simply did not discontinue their treatment when they became very old.

Results from HYVET are probably not enough to answer all questions about treatment of the very elderly with hypertension. There should be caution to extrapolate results to patients in this age group who are more frail than those in the trial, and also to other antihypertensive drugs than those used in the study. Thiazide diuretics in the very elderly may be more prone to metabolic alterations, such as hypokalemia, which can predispose patients to arrhythmias and possibly sudden death. Results from HYVET support a target blood pressure of 150/80 mmHg, whether further reduction is beneficial still needs to be established.

The percentage of elderly hypertensive patients with blood pressure below 140/90 mm Hg is also around 30% in other studies conducted in Spain [3,6,19] and in other countries [20]. Lloyd-Jones found the prevalence of control to decline with age, especially in women [20]. Patients aged 70 and older are less likely to control hypertension than younger groups [21]. The population we studied was homogenous in age and perhaps for this reason this factor was not significant in the regression model. In Spain, the frequency of control of hypertension in adults is higher than that in our study. It ranges from 36.1 to 41.4% [22-24]. Clinicians may be reluctant to treat older patients as aggressively because of perceived lower benefits or possible increased risk of adverse events. Under certain circumstances treatment might be more aggressive. Thus, we found patients with history of ischemic heart disease more frequently with good control. A French study conducted with non-institutionalized subjects aged 65 years and older also found a higher good control in patients with previous cardiovascular events [25].

The frequency of orthostatic hypotension in hypertensive patients increases with advancing age and with systolic blood pressure. It may be present in 5% to 14.6% of patients with hypertension, although a minority has orthostatic symptoms [26]. It is associated with an increased rate of falls, myocardial infarction, transient ischemic attack and ischemic stroke [27]. Antihypertensive drugs may induce orthostatic hypotension or aggravate it when pre-existent [28]. This condition might affect the prescription of antihypertensive drugs or the adherence of patients to medication and, and thus decrease the optimal control of hypertension in this age group.

Our study is population-based, and it includes both people who live in their home and also in geriatric institutions. However an important number of randomized people had already died when they were contacted or refused to take part in the study. This limits to some extent the extrapolation of our results.

The sample was obtained from an urban municipality, Martorell. So data are valid for this municipality. However, similar results might be obtained in other urban populations in Spain. For rural areas, prevalence of hypertension in this age group might be similar, but awareness, treatment and control may be different because of differences in access to health services.

Although regression models were used to identify independent factors for blood pressure control, it was a secondary endpoint of the study. Sample size calculations were based to estimate prevalence of hypertension. Statistical power might be insufficient to identify some factors. Some factors that might be relevant were not considered, such as dementia because some patients with this condition were excluded. Renal failure is often undetected in primary care [29] and for this reason this variable was not collected.

## Conclusion

The prevalence of hypertension in population aged 80 or more is over 70%. Most of the people are aware of this condition. Even though at the time data were collected there was no clear evidence of the benefits from antihypertensive medication in this age group, most of the patients had pharmacological treatment. Half of the subjects had two or more drug products and the most common treatment included diuretic combined with angiotensin converting enzyme inhibitors. Approximately one third of treated patients had blood pressure below 140/90 mm Hg and patients with heart disease and receiving diuretics had better control of hypertension.

## Competing interests

The authors declare that they have no competing interests.

## Authors' contributions

AA designed the study, analyzed the data and drafted the manuscript, FL and SM coordinated the field work and prepared the databases. PO, TB, MF, BH, LE and JP conducted the field work.

All authors read and approved the final manuscript.

## Pre-publication history

The pre-publication history for this paper can be accessed here:


